# Proximal Methods for Plant Stress Detection Using Optical Sensors and Machine Learning

**DOI:** 10.3390/bios10120193

**Published:** 2020-11-29

**Authors:** Alanna V. Zubler, Jeong-Yeol Yoon

**Affiliations:** Department of Biosystems Engineering, The University of Arizona, Tucson, AZ 85721, USA; avzubler@email.arizona.edu

**Keywords:** abiotic stress, plant disease, fluorescence, hyperspectral imaging, thermography, RGB imaging, smartphone imaging, support vector machine (SVM), artificial neural network (ANN), machine learning

## Abstract

Plant stresses have been monitored using the imaging or spectrometry of plant leaves in the visible (red-green-blue or RGB), near-infrared (NIR), infrared (IR), and ultraviolet (UV) wavebands, often augmented by fluorescence imaging or fluorescence spectrometry. Imaging at multiple specific wavelengths (multi-spectral imaging) or across a wide range of wavelengths (hyperspectral imaging) can provide exceptional information on plant stress and subsequent diseases. Digital cameras, thermal cameras, and optical filters have become available at a low cost in recent years, while hyperspectral cameras have become increasingly more compact and portable. Furthermore, smartphone cameras have dramatically improved in quality, making them a viable option for rapid, on-site stress detection. Due to these developments in imaging technology, plant stresses can be monitored more easily using handheld and field-deployable methods. Recent advances in machine learning algorithms have allowed for images and spectra to be analyzed and classified in a fully automated and reproducible manner, without the need for complicated image or spectrum analysis methods. This review will highlight recent advances in portable (including smartphone-based) detection methods for biotic and abiotic stresses, discuss data processing and machine learning techniques that can produce results for stress identification and classification, and suggest future directions towards the successful translation of these methods into practical use.

## 1. Introduction

New and innovative management techniques are needed to ensure a sustainable future for the agricultural industry as the world population continues to increase. There will be over 9 billion people inhabiting the earth by 2050 [1]. Feeding such a large population is a complex problem that will require the utilization of a variety of ideas and techniques across disciplines. Among these is ensuring maximum crop yields with minimal losses from plant stresses such as drought, lack of nutrients, and disease. If proper attention is not given to the mitigation of these yield losses, several components of food security such as availability and economic access may be affected [2].

Pathogens and biotic stresses have received considerable attention in plant stress studies. About 20–30% of crops are lost due to pests and pathogens globally, with many of these losses occurring almost every growing season [3]. Furthermore, many species of plant pathogens can travel over long distances, whether by wind, water, or human activities such as trade and travel [4]. The distribution of pathogens may also be shifted due to the effects of climate change [5]. The detection of plant diseases is therefore essential not only to implement appropriate disease management strategies and mitigate potential losses but also to monitor changes in pathogen distribution. Most diseases can be detected relatively easily when symptoms are fully developed due to noticeable changes in the plant’s appearance; however, early detection is essential in preventing large yield losses. It is also needful to detect abiotic stresses such as water and nutrient deficiencies at an early stage before damages significantly affect crop yields; furthermore, these stresses will become more prevalent in the agriculture industry due to climate change effects such as drought stress and increased salinity [6], which will produce a need for increased environmental monitoring for more refined management practices.

Bioreceptor-based direct detection methods such as polymerase chain reaction (PCR) [7], enzyme-linked immunosorbent assay (ELISA) [8], and flow cytometry (FC) [9] are widely available for the detection of plant diseases; however, these methods require specialized training and can be time-consuming and labor-intensive. An alternative detection method that can be used both for biotic and abiotic stresses is a simple visual observation by an expert [10], but this technique can be prone to bias, with varying results based on the experience of the evaluator.

Optical techniques hold considerable advantages over the previously mentioned techniques, such as a greater potential for rapid disease detection (with some methods producing results in near-real-time [11]), standardized results that are not subject to individual biases, and the ability to detect both biotic and abiotic stresses. Techniques using proximal (near to the target) sensing methods have been utilizing optical sensors that are becoming increasingly smaller and more portable. Although optical sensors provide greater simplicity in the data collection process, the data itself can be complex and large in size (especially in regard to hyperspectral imaging), requiring the use of sophisticated data processing and statistical methods. Furthermore, images and spectroscopic data are not very specific to particular stresses, as opposed to bioreceptor-based (e.g., chemical ligands, antibodies, nucleic acids, etc.) direct detection methods. Despite these limitations, stress specificity and complex data analysis can still be achieved using machine learning techniques, which can analyze the data provided to find patterns that are specific to the plant stress in question. Many studies have successfully utilized machine learning to interpret optical sensor data for the detection of specific stresses. This review aims to provide an outline of current optical sensor types and machine learning methods used to proximally detect plant stresses.

## 2. Spectral Properties of Plant Tissues

**Figure 1:**
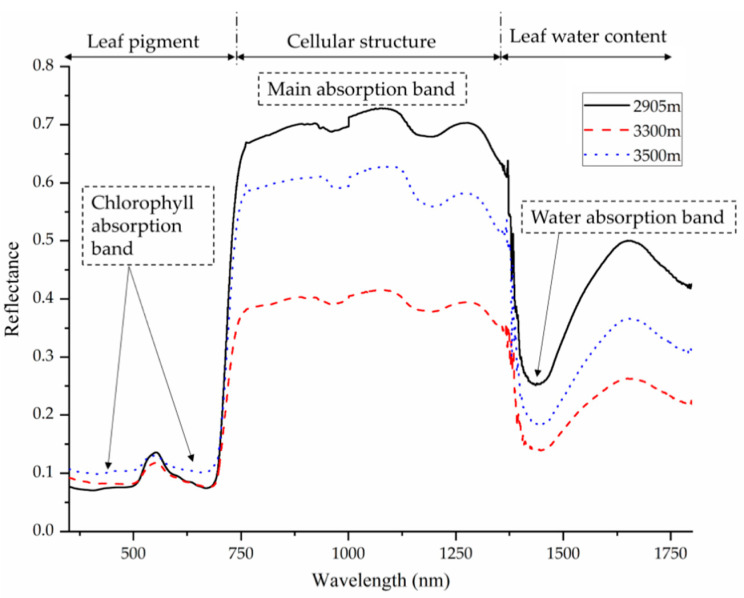
Reflectance spectra of *Quercus aquifolioides* leaves at different altitudes. Vegetation reflectance curves in general typically display this kind of pattern, with low reflectance in the visible region (influenced by leaf pigments), “red edge” connecting the visible and near-infrared (NIR) region, and high reflectance in the NIR region (influenced by cell structure). After 1300 nm, reflectance characteristics are mostly influenced by leaf water content. Reprinted from [12]. ©2020 Zhu et al.

Many physiological and chemical properties of plants influence the way their tissues reflect and absorb light. These properties can change when a plant is subjected to stress and alter the reflectance spectrum of its leaves (Figure 1). 

Chlorophyll is a pigment that is involved in the photosynthesis process. Due to its important role in absorbing light, changes in chlorophyll content resulting from stress will alter the way the plant interacts with light energy. A decrease in chlorophyll content may occur when the plant is subjected to stress, which can be characterized in various ways including an increase of reflectance near 700 nm [12] and decreased reflectance in the 530–630 nm range [13]. Other pigments besides chlorophyll, such as carotenes [14] and xanthophylls [15], can also alter a plant’s reflectance properties. 

**Figure 2:**
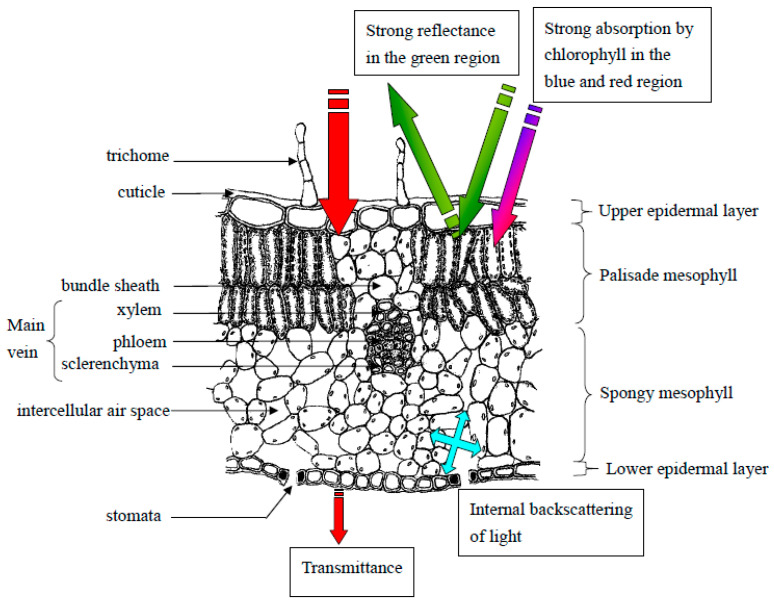
Drawing of a cross-section of a typical leaf with labeled cell types and layers. Basic light interactions with leaf layers are annotated. Reprinted from [21]. ©2008 Liew et al.

In addition to pigmentation, leaf anatomical properties (Figure 2) such as the convexity of epidermal cells [16], surface texture and thickness of the leaf cuticle [17], and high trichome density [18] can be altered under stress and consequently affect a leaf’s spectral properties. For example, exposure to UV radiation can result in changes to chlorophyll content and increased leaf thickness, which can alter chlorophyll fluorescence levels [19]. Reflectance in the 950–970 nm range was found by Peñuelas et al. (1993) to be influenced by cell wall elasticity, which decreases in response to drought stress [20]. 

Small openings on plant leaves (stomata) can also affect leaf properties under stress [21]. These pores are important to regulate moisture and control gas exchange in the leaves; however, microorganisms such as bacteria and fungi can use them to enter and infect a plant. Plants can recognize these pathogens using pathogen- or microbe-associated molecular patterns (PAMPs or MAMPs), which can then trigger stomatal closure to prevent entry [22]. Stomatal closure can lead to an increase in leaf temperature, which can be detected in the infrared region of the electromagnetic spectrum.

The biochemical properties of leaves, such as cellulose, hemicellulose, lignin, protein, sugar, and starch can also change under various stresses and affect the reflectance properties of leaves [23]. For example, salt stress can result in spectral changes by damaging leaf mesophyll cells and altering polysaccharide and lignin composition in the cell wall [24]. Leaf water content can also influence reflectance spectra as light absorption in the infrared region (>1300 nm) is primarily due to water absorption [25].

## 3. Sensors and Data Collection

**Figure 3:**
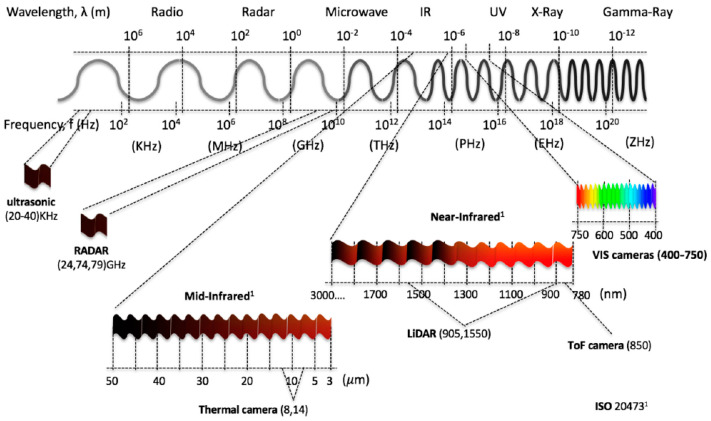
Ranges of the electromagnetic spectrum that are utilized by various sensor types. Useful wavelengths for plant stress detection tend to be in the ultra-violet (UV), visible, and NIR ranges. Reprinted from [27]. ©2019 Rosique et al.

A variety of optical sensors have been used to evaluate plant health, including hyperspectral, multispectral, thermal, and fluorescence sensors (Table 1). The reflectance data collected by these devices can be represented using images acquired by imaging techniques or spectral graphs produced using spectroscopic methods. An important element that can affect a device’s success in stress detection is the sensor’s sensitivity to areas in the plant’s reflectance spectrum that are altered by biotic and abiotic stresses. Generally, the most sensitive region in the electromagnetic spectrum for evaluating plant health is the visible region [26], but other regions can also be influenced by stress. A diagram displaying various wavelength regions in the electromagnetic spectrum is presented in Figure 3 [27].

### 3.1. Hyperspectral Imaging

Hyperspectral imaging utilizes both imaging and spectroscopy methods to produce multi-dimensional data. Spectral information for a wide range of individual wavelengths is assigned to every pixel in an image [28]. Rather than collecting spectra from an entire image or an entire plant leaf, where spectra from the stressed and unaffected areas are mixed together, hyperspectral imaging can provide more sophisticated data that can isolate spectra only from the affected area and identify specific imaging patterns and characteristics. This method has become increasingly popular for plant phenotyping and stress detection in agriculture [29,30,31] and has been used to identify plant responses to both abiotic and biotic stresses, such as drought stress in maize [32] and barley [33], yellow rust [34] and powdery mildew [35] in wheat, salt stress in okra [36], and Black Sigatoka disease in banana plants [37]. 

Hyperspectral imaging for plant status evaluation typically uses a wavelength range of about 250–2500 nm, i.e., UV (ultraviolet), visible, and NIR (near-infrared), with the most important areas in the visible and NIR ranges [38]. Other areas of the spectrum are still being explored in terms of their capability for plant stress detection. For example, Brugger et al. (2019) used hyperspectral imaging in the UV range to detect salt stress in barley [39]. Due to the sensors’ ability to detect a wide range of wavelengths in the electromagnetic spectrum, many possibilities remain for evaluating new combinations of wavelengths for plant stress detection.

The data acquired using hyperspectral techniques are often used to compute and create vegetation indices (VIs). VIs are computed using ratios and combinations of reflectance measurements at a few specific wavelengths and have been used extensively for plant stress monitoring [40,41,42]. In addition to VIs, hyperspectral data can be used to develop spectral disease indices (SDIs) with the purpose of discriminating between specific plant diseases [43] (Table 2). Some examples include indices for detecting powdery mildew in wheat [44] and sugar beet [45], cercospora leaf spot in sugar beet [45], leaf rust in wheat [46], and myrtle rust [47]. Notable vegetation indices include the normalized difference vegetation index (NDVI) [48], water index (WI) [49], and photochemical reflectance index (PRI) [50]. The vast amount of spectral data that is collected using hyperspectral imaging provides great potential in developing new VIs and SDIs for the detection of highly specific plant stresses.

**Figure 4:**
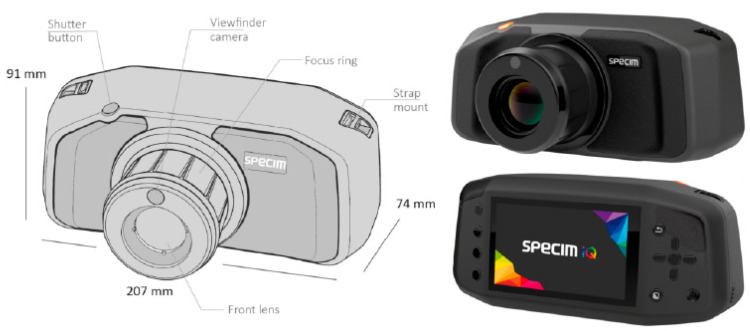
SpecimIQ miniature hyperspectral camera (Specim Ltd., Oulu, Finland). Reprinted from [52]. ©2018 Behmann et al.

The main advantages of hyperspectral imaging include its robustness and ability to provide a large amount of data for analysis; however, this can result in instruments being relatively expensive. In addition, traditional hyperspectral imaging sensors can be bulky and large, which limits their portability and range of applications; however, the development of handheld spectroradiometers and small hyperspectral cameras (Figure 4) has largely addressed this problem. While these instruments typically have a more limited spectral range than a standard hyperspectral sensor, they have the capacity to be used with real-time detection applications [51,52]. Spectroradiometers are unable to capture hyperspectral images; however, they have been used in many studies to detect plant stresses, such as peanut leaf spot disease [53] and powdery mildew in barley [52]. 

Hyperspectral imaging sensors have become increasingly smaller and less expensive; however, considerable progress still remains to create a device that costs less than a few hundred U.S. dollars. Currently, the cost of these cameras is in the thousands of U.S. dollars, which can make them cost-prohibitive to many. Future advances in imaging technology over the coming years should be able to produce a hyperspectral camera or spectrophotometer that is cheaper and more accessible.

### 3.2. Multispectral Imaging and Spectroscopy

Multispectral techniques utilize data from ranges of wavelengths, rather than hundreds of individual wavelengths or narrow wavebands as demonstrated in hyperspectral techniques. A few wavelengths or wavebands of interest can be chosen for incorporation into a device that uses either imaging or spectroscopic techniques. Multispectral imaging involves data collection using a camera or other sensing device to produce image data in specified wavelength or waveband regions, while multispectral spectroscopy produces spectral data for specified wavebands. Both multispectral imaging and multispectral spectroscopy have been successfully used to identify plant stresses; for example, multispectral imaging was used to detect leaf spot disease in oilseed rape [54], gray mold in tomato leaves [55], and nutrient deficiencies in tomato plants [56], while multispectral spectroscopy was used to detect nitrogen deficiency stress in maize [57], drought stress in tomato plants [58], and nitrogen deficiency in canola plants [59]. Multispectral techniques offer more affordable sensors than their hyperspectral counterparts; however, they do not provide as much information about the plant and its environment due to the broader wavebands. Nevertheless, other advantages multispectral methods have are their portability and flexibility, which can aid in the creation of customized devices. Band-pass filters could be used in conjunction with a camera or other imaging device to acquire data in desired spectral ranges at a low cost. Recent modifications in smartphone cameras now permit the capture of NIR wavelengths; Chung et al. (2018) utilized an 800 nm high-pass filter attached to a smartphone to acquire both NIR and red images towards detection of plant stress [60].

### 3.3. RGB Imaging

RGB (visible or red-green-blue) imaging employs sensors that utilize the red, green, and blue regions of the spectrum to produce image data (which is the standard working principle of digital cameras). The wavelengths captured are approximately 400–499 nm for blue light (maximum at 475 nm), 500–549 nm for green light (maximum at 520 nm), and 550–750 nm for red light (maximum at 650 nm) [38]. In this sense, RGB imaging may be considered as a special case of multispectral imaging. However, as RGB imaging data are typically acquired using a digital camera or smartphone while multispectral imaging requires more specific equipment or instrumentation, they are typically treated separately. 

**Figure 5:**
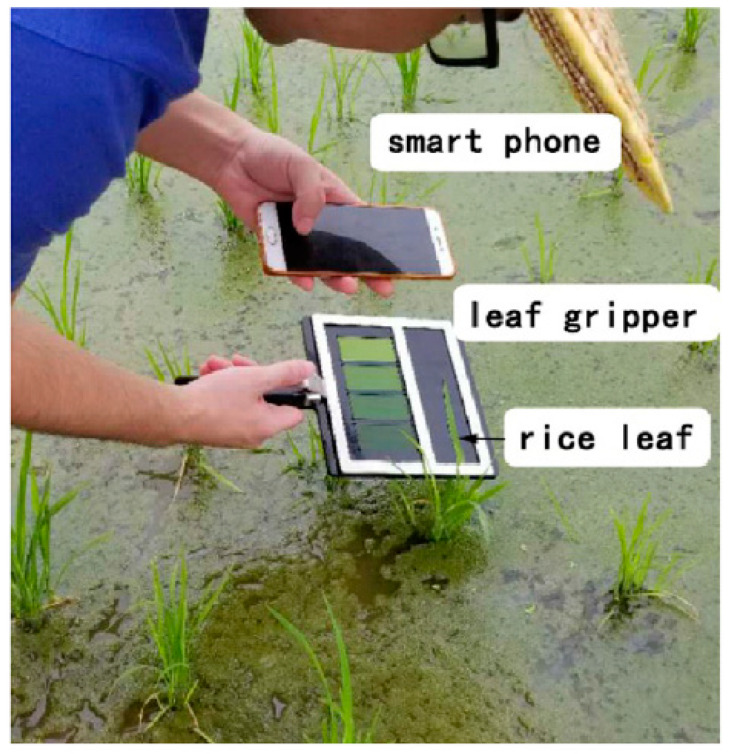
Smartphone being used to evaluate the leaf color of rice plant leaves, which is applicable in detecting nitrogen deficiencies. Reprinted with permission from [65]. ©2020 Elsevier.

The main advantages of RGB imaging are its affordability and small, portable sensor size. RGB image sensors are already present on smartphones and have been used to successfully evaluate plant stresses (Figure 5), such as iron deficiency chlorosis in soybean [11], various nutrient deficiencies in black gram [61], early and late blight in potato plants [62], and biotic stresses in wheat [63]. Furthermore, RGB imaging (especially with smartphones) does not require much technical expertise on the user’s side since they typically make use of commonly used devices such as digital cameras and smartphones. Smartphones also have enough computing power to process the captured data, which enables rapid assessments of plant stresses. However, many factors can complicate RGB data, such as lighting, environmental conditions, time of day, and spectral resolution [64,65]. Illumination is a particularly important concern in terms of field applications since it can vary greatly depending on the season and weather conditions. Diseases with various symptoms and complex image backgrounds can create further complications in processing the data; however, many of these difficulties can be overcome using image processing and machine learning techniques [11].

### 3.4. Thermal Imaging/Thermography

**Figure 6:**
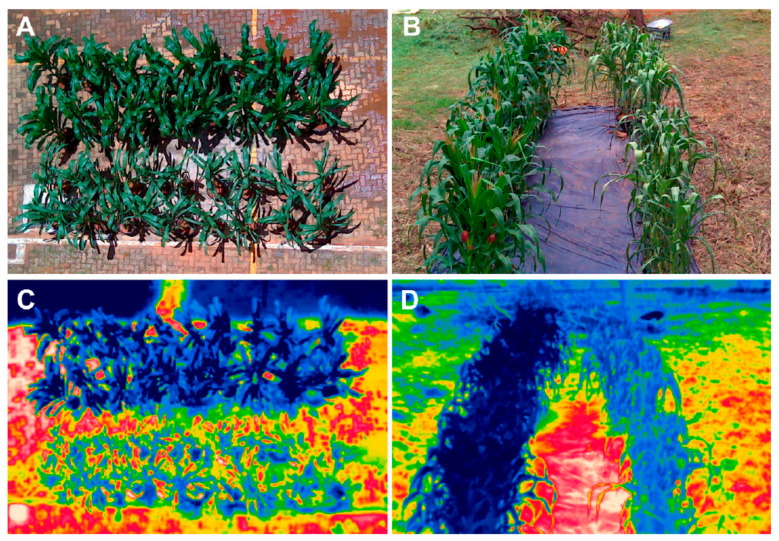
Thermographic image data used to evaluate drought stress in maize plants. In (**A**,**C**) the top row consists of well-watered plants, while the bottom row is drought-stressed. Similarly, in (**B**,**D**), well-watered plants are in the left row, while drought-stressed plants are in the right. Reprinted with permission from [68]. ©2019 Casari et al.

The main difference between thermography and other methods is its measurement of emitted radiation from an object, rather than reflected radiation [66]. Thermal cameras detect radiation in the infrared wavelength range, with the resulting measurements being displayed as false-color images (Figure 6) where the pixels contain the temperature values. Thermographic methods for plant stress detection primarily exploit changes in surface temperature being a notable stress symptom. Small openings on plant leaves (stomata) that control water loss from transpiration may close under stress, causing the temperature of the plant to increase [67]. Thermography has been used to detect a variety of biotic and abiotic stresses, such as *Aspergillus carbonarius* infection in grapes [66], drought stress in maize [68], apple scab disease [69], and drought stress in sesame plants [70].

Thermography is a relatively simple method that can be incorporated into systems designed for the rapid detection of plant stress. Thermal cameras are often very portable, and attachments have been developed that can be used with smartphones. Among these is the FLIR One, which was used by Petrie et al. (2019) to assess the water status of grapevines [71]. However, thermographic methods are highly affected by varying environmental conditions [70], which may make them more applicable in controlled environment applications rather than an open field. Furthermore, thermography lacks specificity and therefore provides a more general solution to plant stress detection. It is recommended to combine thermography with other methods when specific diseases need to be identified since this method is not able to distinguish between different stresses and diseases on its own [69].

### 3.5. Fluorescence Spectroscopy

The above-mentioned imaging methods (hyperspectral, multispectral, and RGB imaging) quantify the attenuations of incident light by the samples (plant leaves in this case) over the range of wavelengths, i.e., spectrophotometric detection. Since many components in plant leaves exhibit colorations and subsequently spectrophotometric responses, the resulting spectrophotometric images tend to be quite complex. Fluorescence-based methods can fix this issue, as only a small number of components in plant leaves exhibit fluorescence. Fluorescent molecules (e.g., chlorophyll, fluorescent dyes, etc.) absorb light at a specific wavelength (excitation) and emit at a specific, longer wavelength (emission), thus incident and emitted light can be separated. The two main types of fluorescence emitted by vegetation are blue-green fluorescence (400–600 nm) [72] and chlorophyll fluorescence (650–800 nm) [73]. The latter can be useful in evaluating photosynthetic activity, which can decrease under pathogenic stresses [74].

Although several techniques are available, two major methods for acquiring fluorescence data in plants are pulse-amplitude modulation (PAM) of the measuring light and continuous illumination [75]. Pulse-amplitude modulation devices use a pulsed measuring light source, an actinic light source, and a saturating light to obtain fluorescence signals [76]. In contrast, light is not pulsed when continuous illumination is utilized.

Fluorescence can be measured as a spectrum from a single point in time [77], or the change in fluorescence over time can be monitored (chlorophyll fluorescence kinetics). The basic principle behind chlorophyll fluorescence techniques is a lowered rate of photosynthesis from stresses and subsequent dissipation of chlorophyll fluorescence [78]. Fluorescence kinetics measurements require the use of dark adaptation, which consists of placing a plant (or the part of the plant to be measured) in the dark for a certain period of time before fluorescence measurements are taken. Dark adaptation allows for the measurement of the minimum level of fluorescence [79], which is a fundamental value in kinetics analysis since it provides a baseline for the other fluorescence measurements taken after the excitation light has been introduced. Plants are usually dark-adapted for a period of 30 min [80,81,82]. Regardless of whether dark adaptation is utilized or not, it is essential to give plants the necessary time to adapt to light conditions before measurements (for kinetics applications or standard spectra) are taken. 

Fluorescence ratios are often used to analyze fluorescence data (both images and spectra) for evaluating plant stresses. Common ratios involving UV-induced (320–400 nm) fluorescence include F440/F520, F440/F690, F440/F740, and F690/F740; F440/F690 and F440/F740 are particularly useful for early stress detection applications (F represents fluorescence and the numbers represent emission wavelengths) [83]. Bürling et al. (2011) used red/far-red and blue/green amplitude ratios acquired from spectral signatures to differentiate between nitrogen deficiency, leaf rust, and powdery mildew stresses [84]. Although the ratios mentioned above are relatively well-established in fluorescence research, there is still room for exploration in determining other ratios that could be used to process data.

**Figure 7:**
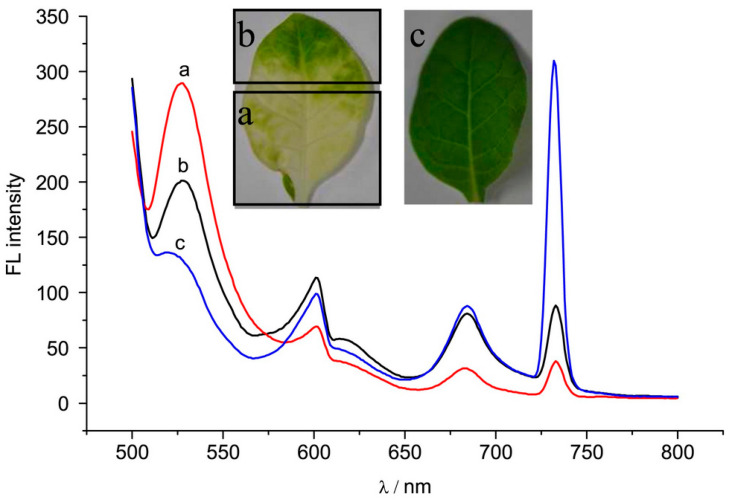
Fluorescence emission spectra of leaves excited at 488 nm. (**a**) chlorotic part of tobacco leaf infected by cucumber mosaic virus; (**b**) green part of tobacco leaf infected with cucumber mosaic virus; (**c**) a healthy tobacco leaf. Reprinted with permission from [77]. ©2016 John Wiley and Sons.

Fluorescence spectroscopy can identify the location and amount of a specific component from the sample through applying a narrow-range excitation light and detecting a narrow-range emission from such component. Figure 7 is an example of fluorescence spectroscopy, where the plant leaves are excited at 488 nm (blue color) and a spectrum with wavelengths of >500 nm (green and red colors) is collected. There is a clear difference between the healthy and virus-infected plant leaves. Fluorescence spectroscopy has been used in many other studies to detect both biotic and abiotic stresses, including drought stress in passion fruit [80]; nutrient stresses in maize [81], tomato [81], and rapeseed [82] crops; and citrus canker on grapefruit plants [85]. 

Fluorescence spectroscopy has advantages such as simplicity of use, low cost, and an ability to be incorporated into hand-held devices for screening applications [79]. In addition, the use of laser light as an excitation light source can be more reliable than other optical methods, as excitation exactly at the sample’s peak excitation wavelength can generate stronger and more specific fluorescent emission (as opposed to passive measurements) [86]. Fluorescence data can be collected across multiple wavelengths, which can provide more information than fluorescence captured at a single targeted wavelength. However, fluorescence spectroscopy alone still lacks specificity [85] because changes in fluorescence can be indicative of a wide variety of stresses. Therefore, it is necessary to combine this method with others if discrimination between specific stresses is to be achieved. Another challenge related to chlorophyll fluorescence kinetics is the reduction of fluorescence intensity over time (photoquenching or photobleaching); however, Saleem et al. (2020) were able to mitigate its effects by measuring fluorescence spectra quickly (about 15 s) after the excitation light was introduced [85]. 

### 3.6. Fluorescence Imaging

**Figure 8:**
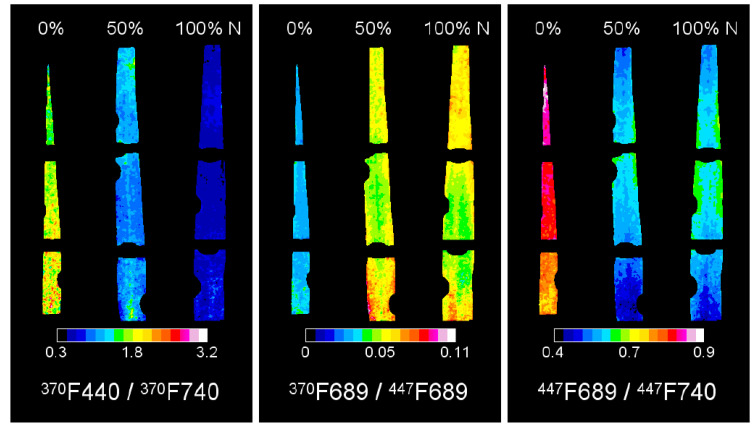
Fluorescence ratios of barley leaves with nitrogen (N) deficiencies of varying severity. Reprinted from [90]. ©2014 Konanz et al.

Fluorescence imaging utilizes a camera to obtain images of fluorescence (Figure 8). It is considered an improvement over spectroscopy since it obtains fluorescence data with higher dimensions, which can provide more information than single spectra. Rather than collecting a spectrum from an area of interest (i.e., fluorescence spectroscopy), fluorescence imaging can isolate the area of interest from that of non-interest. For example, Su et al. (2019) used fluorescence imaging to successfully discriminate crops from weeds [87]. One category of continuous fluorescence imaging is multicolor fluorescence imaging, which typically uses UV excitation light and collects fluorescence data from multiple bands, such as red (F680), far-red (F740), green (F520), and blue (F440) [83]. Multicolor fluorescence imaging is conceptually similar to multispectral imaging since only certain fluorescence wavebands are collected and combined to produce the image. Fluorescence imaging can also be used with dark adaptation and chlorophyll fluorescence kinetics applications.

Fluorescence imaging has been used in many studies to detect both biotic and abiotic stresses, such as herbicide stress in soybeans [88], cold stress in tomato seedlings [89], and biotic and abiotic stresses in barley, grapevine, and sugar beet [90]. A relatively simple and portable option for fluorescence image acquisition could consist of a smartphone and band-pass filters (as demonstrated in [91]); however, it is currently difficult to find methods with this type of setup for plant stress applications.

One advantage of fluorescence-based techniques is their sensible cost of equipment [92]; however, they do not always produce a clear distinction of healthy and diseased plant tissues at the early stage of a disease, so additional methods may be necessary to complement fluorescence for early disease detection [93]. Fluorescence-related methods could benefit from an increased sensitivity that could allow them to be used for stress discrimination applications rather than simple stress identification.

### 3.7. Combination of Sensors

Combining two or more of the methods mentioned above can provide more information on plant health as opposed to using just one method. The merging of data from multiple sensors has been successful in plant stress detection; for example, Moshou et al. (2011) used a combination of multispectral and hyperspectral imaging to detect yellow rust in wheat [94]. Many advantages are offered by using multiple sensors, including higher accuracy and decreased sensitivity to changes in the environment [94]; however, a major challenge is the merging of different data types. One possible solution is a discriminant analysis, which was used by Berdugo et al. (2014) to combine thermographic, hyperspectral, and chlorophyll fluorescence data to differentiate between cucumber mosaic virus, green mottle mosaic virus, and powdery mildew in cucumber plants [95]. Sensor combination shows great potential in producing accurate, highly specific data; however, more research is needed in methods to combine data from multiple sources with different properties and work with larger amounts of data [95]. Machine learning could be a pivotal tool in analyzing such combinatory sensor data.

A variety of sensors have been used to identify stresses in agricultural crops [96,97,98,99,100,101]; however, their detection capabilities could be greatly enhanced by incorporating machine learning techniques, which are discussed in the following sections.

## 4. Machine Learning for Data Processing

**Figure 9:**
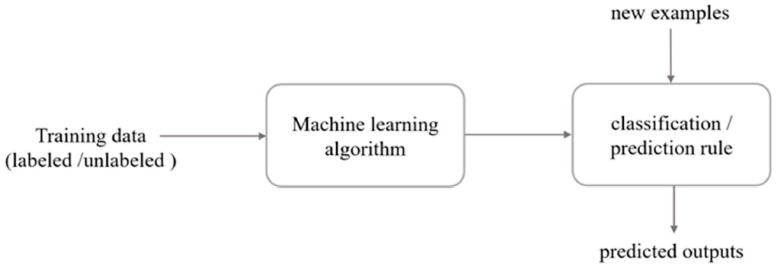
A simplified machine learning pathway. Reprinted from [104]. ©2018 Liakos et al.

Machine learning has opened possibilities for new data analysis methods in a myriad of fields, including medicine, environmental science, and economics. Fundamentally, machine learning employs techniques to learn from the given data without providing explicit programming commands [102], which can result in the detection of new patterns that may otherwise be overlooked using traditional analytical methods. Major processes in a machine learning procedure include data acquisition and storage, preprocessing, classification, and trait extraction [103]. Figure 9 [104] outlines a simplified pathway for machine learning data analysis methods.

Machine learning is advantageous in agriculture-related fields because it can detect patterns using simultaneous combinations of multiple factors instead of examining traits individually [102]. The use of multiple factors is important due to the frequently high complexity of the environment surrounding plants, where variables such as changing light intensity, direction, and leaf angle can alter results. Machine learning can be used not only for classification purposes but also for pre-processing steps such as feature extraction and dimensionality reduction.

The assessment of plant health includes stress identification, discrimination, and quantification. Identification involves looking for symptoms (early or late) of a specific stress, discrimination consists of both identifying a specific stress and separating the symptoms from those of other stresses, and quantification is a measurement of the severity of the stress. Machine learning has been utilized for all these applications, as outlined in Table 3.

The selection of a machine learning method or pathway depends on the specific problem being addressed; as such, there is currently no specific approach that can be recommended for all applications. The following sections will provide an overview of machine learning data processing techniques that have been used for various agricultural applications. 

### 4.1. Preprocessing

Data preprocessing is essential to ensure the accuracy and reproducibility of classification results [105]. Preprocessing consists of one or more operations that aim to improve the performance of the classification algorithms by providing data in a more accessible and normalized format. Image preprocessing techniques may include image cropping, background removal, contrast enhancement, image thresholding, noise removal with filters, clustering, and principal component analysis (PCA) [102]. Although this section deals mostly with imaging techniques, spectral data may also be processed using some of the listed methods, such as PCA. Outlined below are some preprocessing steps that are commonly applied to imaging data.

#### 4.1.1. Color Space Conversion

**Figure 10:**
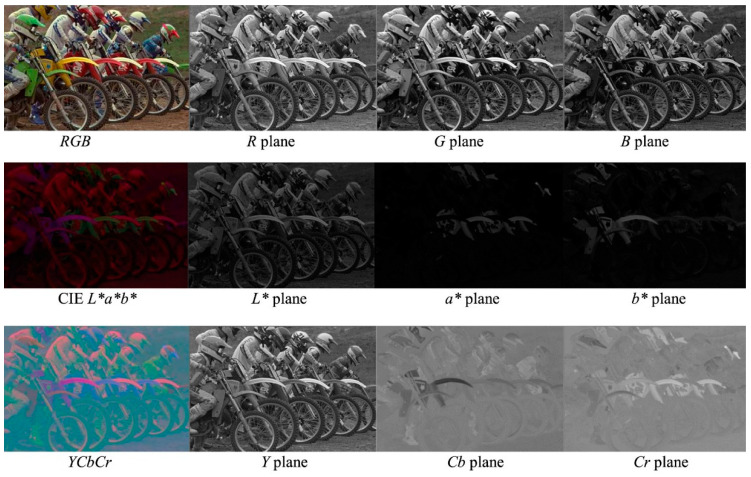
An RGB (visible or red-green-blue) image represented using other color spaces. Adapted with permission from [109]. ©2018 John Wiley and Sons.

Color space conversion is a data processing technique that can be used with RGB images as another way to represent color. Color spaces can be used to acquire additional color features from images to aid in feature extraction and image classification. Several studies have used features obtained from color space conversion to process RGB data for plant stress detection, including L*a*b* (L* = lightness from black to white, a* = from green to red, and b* = from blue to yellow) to detect bacterial blight, fruit spot, fruit rot, and leaf spot in pomegranate plants [106]; HSI (hue, saturation, intensity) to detect early scorch, late scorch, cottony mold, ashen mold, and tiny whiteness in plants [107]; and YCbCr (Y = luma component; Cb and Cr = blue- and red-differences of chroma components) to detect diseases in soybean [108]. A few alternative color spaces are outlined in Figure 10 [109].

#### 4.1.2. Dimensionality Reduction

Dimensionality reduction is a process that aims to provide a more compact representation of data while preserving as much information as possible. A common method for dimensionality reduction is principal component analysis (PCA), which geometrically projects data onto lower dimensions (principal components) that act as feature summaries [110]. PCA can combine dependent (or highly correlated) variables into a common variable while minimizing the loss of information. By doing so, the dimensionality of data can be reduced. The first principal component (PC1) is evaluated from the data set. Then PC2 is evaluated from the remainders, and the process is repeated, e.g., PC3, PC4, etc. The principal components (PCs) represent data variances, and these can be plotted in 2D or 3D plots (in the case of two or three PCs) known as PCR score plots. 

All PCs can also be fed into the various machine learning models as a pre-processing step of dimensionality reduction. PCA has been used in many studies as an important preprocessing step to manage both imaging and spectral data. For example, PCA was used in an image preprocessing pipeline by Lu et al. (2017) to aid in acquiring feature maps [111]. While better dimensionality reduction methods have recently emerged, e.g., linear discriminant analysis (LDA) that can maximize the class separation, PCA is often preferred over the recent methods as an unbiased dimensionality reduction method. PCA can be a valuable tool to aid in data interpretation, but one disadvantage of this method is its ability to be influenced by outliers in the data [112].

#### 4.1.3. Segmentation

**Figure 11:**
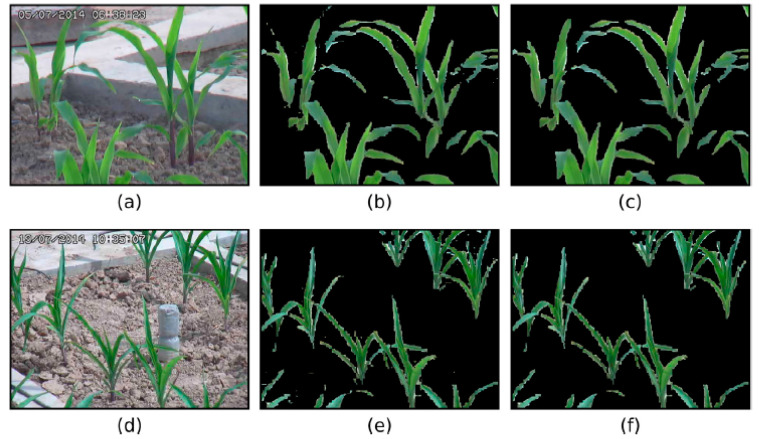
A visualization of the image segmentation process. (**a**,**d**) are the original samples of well-watered and drought-stressed maize plants. (**b**,**e**) are preliminary segmentation images acquired using RGB pixel values and linear support vector machine (SVM), while (**c**,**f**) are the images denoised using the mathematical morphology method. Reprinted with permission from [115]. ©2017 Elsevier.

Image segmentation is a process that can organize an image into key areas, such as the object and its background. This technique is useful in agricultural applications due to its ability to reduce errors or misclassifications resulting from noise in the background. Notable methods include clustering-based approaches such as k-means, which can be useful in identifying stressed areas of a plant in an image [107]. Disease detection applications may require other techniques such as pixel removal and masking [113]. For example, Ma et al. (2018) used excess red index (ExR), H from the HSV (hue, saturation, value) color space, and b* from the L*a*b* color space to discriminate between disease spots and background in images [114]. An example of segmentation being used to separate plants from the background of an image is demonstrated in Figure 11 [115].

#### 4.1.4. Feature Extraction

Feature extraction can be used to express data in a format that is more accessible to machine-learning algorithms [105]. It consists of reducing redundant data and collecting a set of extracted features; for images, available techniques include Global Color Histogram [116], Local Binary Patterns [117], and Color Coherence Vector [118]. Features can include color-related characteristics such as the variance of color channels and texture features such as contrast and channel homogeneity [114]. These acquired features are then analyzed using the classification algorithms.

### 4.2. Machine Learning Algorithms for Classification

Once the necessary preprocessing steps are complete, the data can be fed into a machine learning algorithm for classification. These algorithms attempt to find patterns in data to use in assigning classes (e.g., stressed vs. healthy) to unlabeled data [29]. Machine learning algorithms can be divided into supervised, weakly-supervised, and unsupervised categories, all of which can be used for classification [119,120]. The major difference among these algorithms is supervised learning involves the use of labeled training data to predict the labels of testing data; weakly-supervised learning can use smaller datasets, coarse labels, or misclassified labels for training, and unsupervised learning uses only unlabeled data [120]. One of the most prominent examples of unsupervised learning is clustering algorithms, which create clusters consisting of samples with similar traits [121]. 

Many machine learning algorithms have been used in agriculture to classify data; however, the most common methods include artificial neural networks (ANNs) [122] and support vector machines (SVMs) [29]. This review will primarily focus on SVM, ANN, and deep learning methods; however, other algorithms such as random forest [123] have been successfully used for plant stress identification applications. 

Machine learning techniques can be very robust classifiers, yet one drawback is their tendency to overfit the data (especially when the data set is small), which results in incorrect classifications. In addition, machine learning can be time-consuming, especially when large image files are involved. Both issues, however, can be mitigated using some of the following processes. One method that has been used to mitigate overfitting errors in image classification is data augmentation, which consists of slightly distorting the images using techniques such as rotation [124], mirroring [125], and color variation [126]. If data augmentation and image manipulation are deemed necessary in the data processing pathway, they must be performed before running the data through the classification algorithm.

#### 4.2.1. Support Vector Machine (SVM)

**Figure 12:**
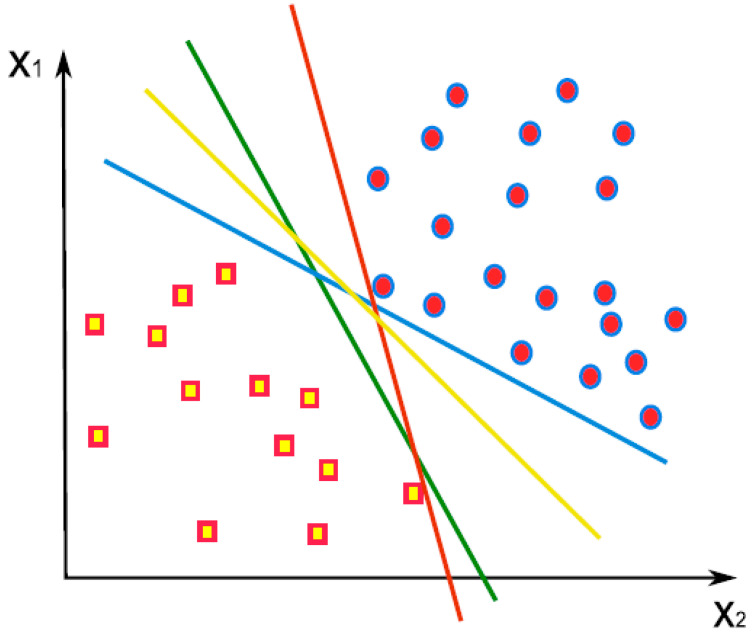
A decision boundary is established (a line for 2D data) for the data reduced in two dimensions, e.g., through PCA. Many boundaries can be drawn, but the best separation will need to be determined. For 3D, the decision boundary is a plane. For dimensions higher than three, the decision boundary is a hyperplane. Reprinted with permission from [127]. ©2020 Elsevier.

SVM is a supervised learning method, i.e., requiring training data set to identify classes of unknown data. Let us assume a simple case that most (e.g., >90%) of the training data set can be reduced to two dimensions through dimensionality reduction methods such as PCA. These data can be plotted on a 2D coordinate system (i.e., PCA score plot). With known classes (e.g., stressed vs. healthy) of the data, it is possible to draw a line that can best separate all of the data into two classes; this line is called a decision boundary (demonstrated in Figure 12 [127]). The procedure can also be used for three or more dimensions of data, where the boundary becomes a plane for three dimensions or a hyperplane for dimensions higher than three. It may be necessary to use about 10 principal components from PCA, but this dimension number is still substantially small compared to the dimensions of the raw data, which could range from hundreds (for spectra) to millions (for images). Testing data is fed into the same data processing pathway as the training data, and the decision boundary formed during training determines the class of testing data. While SVM is inherently a linear method, non-linear separation is also possible using non-linear kernels. Classification into multiple classes is also possible using multiple decision boundaries.

SVMs are one of the most common machine learning algorithms used in agriculture applications. They have been successfully used in many studies relating to plant stress detection, such as identifying Huanglongbing (HLB; also known as citrus greening disease) and nutrient stresses in citrus leaves [100], as well as rating the severity of iron deficiency chlorosis in soybeans [11]. A similar method, relevance vector machine (RVM), was used to identify stripe rust and powdery mildew in wheat [63]. 

While SVM is simple in principle and works quite well with very high dimensions of data (such as spectra and images), it does not explain how close or far away errors are from the true class identification. This is particularly problematic when the data set is noisy, where a distinct decision boundary cannot be determined clearly.

#### 4.2.2. Artificial Neural Network (ANN) 

An artificial neural network (ANN) is a machine learning model that mimics the function of a biological neural network [128]. The basic architecture consists of artificial neurons that process several inputs weighted according to their importance and produce a corresponding output [124]. 

**Figure 13:**
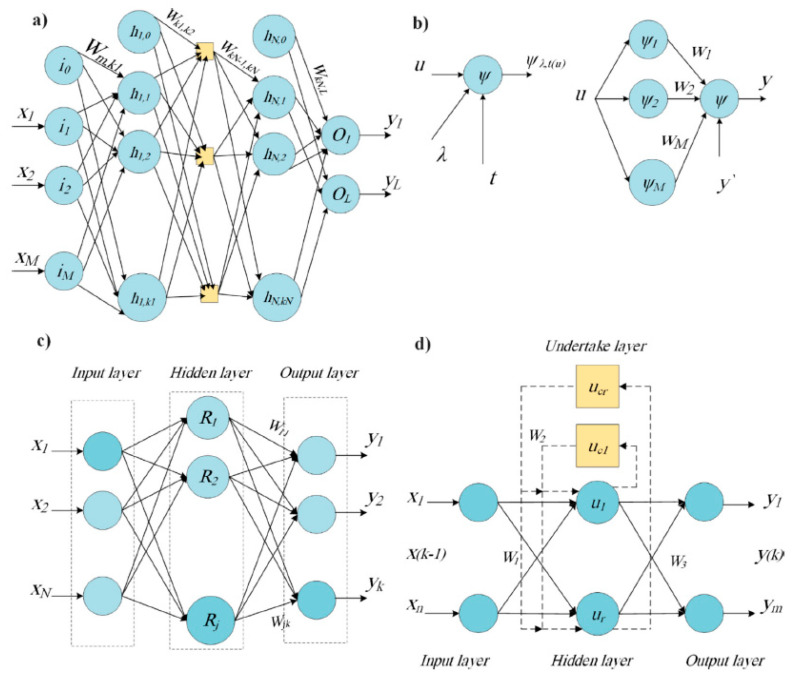
Structures of four types of ANNS: (**a**) multilayer perceptron, where *x_i_* represents inputs, *O_i_* represents output neurons, *h_i_* represents hidden layer neurons, and *w_i_* represents the weights between neurons; (**b**) wavelet, where Ψ represents the wavelet function, *t* represents the translation coefficient, λ represents the dilation coefficient; (**c**) radial basis function, where *R_i_* represents the radial basis function; and (**d**) Elman, where *u_i_* represents components in the hidden and undertake layers. Reprinted with permission from [133]. ©2019 Elsevier.

ANNs have been used successfully in many studies for the identification and classification of various plant stresses. These include detecting powdery mildew and soft rot in zucchini [129], classifying biotic stresses in pomegranate [106], detecting orange spotting disease in oil palm [130], and identifying crown rot in wheat [131]. A major advantage of ANNs is their ability to be used without specialized knowledge on the data and its interpretation; however, disadvantages include being prone to overfitting and requiring greater amounts of computational resources [132]. Several types of ANNs exist, some of which are outlined in Figure 13 [133].

#### 4.2.3. Deep Learning 

Deep learning is a subcategory of machine learning that utilizes ANNs and consists of more advanced models with multiple layers (“deep” indicates the depth of layers). A common model used in agriculture is the convolutional neural network (CNN), which performs convolutions on data for image classification [134]. CNNs and their variations have been frequently used in plant stress studies that utilize machine learning, such as detecting the breaking virus in tulips [135], identifying potato Y virus [136], gauging the severity of apple black spot [119], classifying biotic stresses on cucumber leaves [114], and rating the severity of biotic stresses on coffee leaves [126]. Pretrained CNN models such as GoogleLeNet [137], AlexNet [114], ResNet [138], and VGG [139] have also been used. For instances where an extensive array of training data is required, many studies utilize databases such as PlantVillage [140] and the Wheat Disease Database [141], both of which have been used in conjunction with deep learning models.

One advantage of deep learning techniques is that they work well with raw data [142], which therefore cuts down on time spent in data preprocessing (color space conversion, dimensionality reduction, segmentation, and feature extraction). In addition, feature extraction is sometimes performed in the deep learning model without the need for an outside processing step [143]. However, a major disadvantage is a need for large datasets (often numbering in the thousands [139,144]) to produce accurate results [111].

**Table 3:** Machine Learning Algorithms Used for Plant Stress Detection.

Purpose	Data Type	Plant	Stress	Algorithm	Accuracy	References
Identification	Fluorescence imaging	Zucchini	Soft rot	ANN	100%	[129]
				SVM	90%	
				Logistic regression analysis	60%	
			Powdery mildew	ANN	71.2%	
				SVM	48.1%	
				Logistic regression analysis	73.1%	
Identification	Hyperspectral	Oil palm	Orange spotting disease	Multilayer perceptron neural network	-	[130]
Identification	Hyperspectral	Wheat	Crown rot	ANN	74.14%	[131]
				Logistic regression	53.45%	
				K nearest-neighbors	58.62%	
				Decision trees	56.90%	
				Extreme random forest	58.62%	
				SVM	50%	
Identification	RGB images	Tulip	Tulip breaking virus	Faster R-CNN	86% *	[135]
Identification	Hyperspectral	Potato	Potato virus Y	Fully convolutional neural network	92% *	[136]
Classification	RGB images from smartphone	Wheat	Powdery mildew, stripe rust	RVM	88.89%	[63]
				SVM	77.78%	
Classification	RGB images from database	Pomegranate	Fruit spot, bacterial blight, fruit rot, leaf spot	Multilayer perceptron	90%	[106]
Classification	RGB images	Cucumber	Anthracnose, downy mildew, powdery mildew, target leaf spots	Deep CNN	92.2%	[114]
				SVM	81.9%	
				AlexNet	92.6%	
				Random Forest	84.8%	
Classification	Hyperspectral	Sugar beet	Cercospora leaf spot, sugar beet rust, powdery mildew	SVM	86.42%	[29]
Classification	RGB images from database	Wheat	Powdery mildew, smut, black chaff, stripe rust, leaf blotch, leaf rust	VGG-CNN-S	73%	[141]
				VGG-FCN-S	95.12%	
				VGG-CNN-VD16	93.27%	
				VGG-FCN-VD16	97.95%	
Quantification	Hyperspectral	Barley	Drought stress	Ordinal SVM	67.9%	[33]
Quantification	RGB images from digital camera	Soybean	Iron deficiency chlorosis	Hierarchical SVM-SVM	99.2%	[11]
				Hierarchical LDA-SVM	98.3%	
				Decision tree	99.7%	
				Quadratic discriminant analysis	98.5%	
				Naïve Bayes	98.4%	
				K-Nearest-Neighbors	99.5%	
				Random forest	99.1%	
				Gaussian mixture model	99.4%	
				Linear discriminant analysis (LDA)	98.5%	
				SVM	97.3%	
Quantification	RGB images from database	Apple	Black rot	VGG16	90.4%	[119]
				ResNet50	80%	
Quantification	RGB images from smartphone	Coffee	Leaf miner, rust, brown leaf spot, cercospora leaf spot	AlexNet	84.13%	[126]
				GoogleLeNet	82.94%	
				VGG16	86.51%	
				ResNet50	84.13%	
				MobileNetV2	84.52%	

* Indicates a recall value, not an accuracy value.

## 5. Concluding Remarks

A variety of optical sensing methods and machine learning techniques have been used to recognize both biotic and abiotic stresses, especially plant diseases. One observation is that machine learning is commonly used to process imaging data (especially RGB images), but spectroscopic methods more frequently utilize traditional statistical methods. In the future, machine learning methods could be further incorporated into spectroscopic data analysis pathways. 

Currently, many of the studies mentioned are producing detection results that are specific to just a few plants. Leaf reflectance properties can differ greatly between plant species, so it is difficult to produce results that are generalizable to several plants in different circumstances. The development of more generalized (rather than species-specific) results is likely a future direction in plant stress detection; however, more research is needed to find features and parameters that can lead to such results. Methods such as smartphone imaging, thermography, and fluorescence imaging have the potential to be scaled up to larger-scaled systems to analyze plant canopies in open fields or controlled environments.

Imaging devices (especially multispectral/RGB sensors) have improved in quality and become more compact over recent years. Optical resolutions of recent smartphones’ cameras are comparable to most standalone digital cameras, effectively eliminating the bulk of digital camera markets and only leaving the high-end markets. Sensitivity has also improved dramatically; the white LED flash is rarely necessary with recent smartphones. Computing power and memory have also improved significantly for recent smartphones, which has enabled on-board image processing to become a reality. Cloud storage and computing for remote file management and execution also complements the smartphone’s computing power and memory capacity, allowing for more advanced data processing operations to be performed. Optical zooms (which magnify images mechanically using optical lenses) are possible with recent smartphones, although limited at 2x − 4x at the time of writing. Furthermore, smartphones have the data processing power needed to run machine learning algorithms and thus can provide a rapid, on-site assessment of plant stresses.

The discrimination of specific stresses (especially stresses from specific nutrients) remains a challenge. Discrimination may become more feasible with improvements in the sensitivity of optical devices; however, this increased sensitivity may result in data being more prone to noise from the surrounding environment. Environmental noise could be overcome by the use of image segmentation and machine learning models to help distinguish between noise and the targeted characteristic.

Many improvements are being made with imaging technology and data processing techniques that will enable the development of robust, portable devices for plant stress detection. Although research is still needed in many areas such as the fusion of data from multiple sensors and discrimination between specific biotic and abiotic stresses, current developments have great potential to be deployed as useful tools for the agriculture industry.

## Figures and Tables

**Table 1:** Optical Methods Used for Plant Stress Detection.

Method	Wavelengths	Plant	Stress Type	References
Hyperspectral Imaging	500–850 nm	Maize	Drought stress	[32]
	430–890 nm	Barley	Drought stress	[33]
	350–2500 nm	Wheat	Yellow rust	[34]
	350–1350 nm	Wheat	Powdery mildew	[35]
	380–1030 nm	Okra	Salt stress	[36]
	400–1000 nm	Banana	Black Sigatoka	[37]
	250–430 nm	Barley	Salt stress	[39]
	400–1000 nm	Barley	Powdery mildew	[52]
	325–1075 nm	Peanut	Leaf spot	[53]
Multispectral Spectroscopy	400–1100 nm	Maize	Nutrient deficiency	[57]
	400–980 nm	Tomato	Drought Stress	[58]
	430–870 nm	Canola	Nutrient deficiency	[59]
Multispectral Imaging	365–960 nm	Oilseed Rape	Light leaf spot	[54]
	475, 560, 668, 717, 840 nm	Tomato	Gray Mold	[55]
	550, 660, 735, 790 nm	Tomato	Nutrient deficiency (multiple)	[56]
	620, 870 nm	Poinsettia	Nitrogen content	[96]
	450–950 nm	Wheat	Stripe rust, brown rust, septoria tritici blotch	[97]
RGB Imaging	RGB	Soybean	Iron deficiency	[11]
	RGB	Black Gram	Nutrient deficiency (multiple)	[61]
	RGB	Potato	Early blight, late blight	[62]
	RGB	Basil	Nitrogen stress	[98]
Thermography	7.5–13 μm	Table Grapes	*Aspergillus carbonarius*	[66]
	7.5–13 μm	Maize	Drought stress	[68]
	8–12 μm	Apple	Apple scab	[69]
	8–14 μm	Sesame	Drought stress	[70]
	8–14 μm	Wheat	Drought stress	[99]
Fluorescence Spectroscopy ^1^	650 nm	Passion Fruit	Drought stress	[80]
	635 nm	Maize, Tomato	Nutrient deficiency (multiple)	[81]
	650 nm	Rapeseed	Nutrient deficiency (multiple)	[82]
	405 nm	Grapefruit	Citrus canker	[85]
	337 nm	Wheat	Nutrient deficiency, leaf rust, powdery mildew	[84]
Fluorescence Imaging ^1^	340, 447, 550 nm	Barley, Grapevine, Sugar Beet	Nutrient deficiency, black rot, leaf spot	[90]
	460 nm	Soybean	Herbicide stress	[88]
	620 nm	Citrus	Huanglongbing	[100]
	684, 687, 757.5, 759.5 nm *(emission)*	Cassava	Mosaic virus	[101]

^1^ In fluorescence spectroscopy and fluorescence imaging, excitation wavelengths are shown except noted otherwise.

**Table 2:** Equations and Applications of Vegetation and Disease Indices.

Index Name	Equation ^1^	Application	References
**Vegetation Indices**			
Enhanced Vegetation Index	$EVI=2.5\times \frac{R_{800}-R_{670}}{R_{800}+6.0R_{670}-7.5R_{479}+1}$	Rate of photosynthesis, water stress detection	[41]
Normalized Difference Vegetation Index	$NDVI=\frac{R_{NIR}-R_{RED}}{R_{NIR}+R_{RED}}$	Plant growth and development monitoring	[48]
Water Index	$WI=\frac{R_{900}}{R_{970}}$	Plant water content estimation	[49]
Photochemical Reflectance Index	$PRI=\frac{R_{570}-R_{531}}{R_{570}+R_{531}}$	Photosynthetic efficiency	[50]
**Disease Indices**			
Powdery Mildew Index (Wheat)	$PMI=\frac{R_{515}-R_{698}}{R_{515}+R_{698}}-0.5R_{738}$	Powdery mildew detection in wheat	[44]
Powdery Mildew Index (Sugar Beet)	$PMI=\frac{R_{520}-R_{584}}{R_{520}+R_{584}}+R_{724}$	Powdery mildew detection in sugar beet	[45]
Cercospora Leaf Spot Index	$CLS=\frac{R_{698}-R_{570}}{R_{698}+R_{570}}-R_{734}$	Cercospora leaf spot detection in sugar beet	[45]
Leaf Rust Disease Severity Index 1	$LRDSI_{1}=6.9\frac{R_{605}}{R_{455}}-1.2$	Severity estimation of wheat leaf rust	[46]
Leaf Rust Disease Severity Index 2	$LRDSI_{2}=4.2\frac{R_{695}}{R_{455}}-0.38$	Severity estimation of wheat leaf rust	[46]
Lemon Myrtle—Myrtle Rust Index	$LMMR={\left(\frac{R_{545}}{R_{555}}\right)}^{\frac{5}{3}}\times \frac{R_{1505}}{R_{2195}}$	Myrtle rust detection in lemon myrtle	[47]

^1^R represents the measured reflectance at the wavelength or waveband specified by the subscript.
